# HCN1 channels reduce the rate of exocytosis from a subset of cortical synaptic terminals

**DOI:** 10.1038/srep40257

**Published:** 2017-01-10

**Authors:** Zhuo Huang, Gengyu Li, Carolina Aguado, Rafael Lujan, Mala M. Shah

**Affiliations:** 1 UCL School of Pharmacy, University College London, London, WC1N 1AX, UK; 2 Departamento de Ciencias Medicas, Universidad de Castilla-La Mancha, 02006 Albacete, Spain

## Abstract

The hyperpolarization-activated cyclic nucleotide-gated (HCN1) channels are predominantly located in pyramidal cell dendrites within the cortex. Recent evidence suggests these channels also exist pre-synaptically in a subset of synaptic terminals within the mature entorhinal cortex (EC). Inhibition of pre-synaptic HCN channels enhances miniature excitatory post-synaptic currents (mEPSCs) onto EC layer III pyramidal neurons, suggesting that these channels decrease the release of the neurotransmitter, glutamate. Thus, do pre-synaptic HCN channels alter the rate of synaptic vesicle exocytosis and thereby enhance neurotransmitter release? To address this, we imaged the release of FM1-43, a dye that is incorporated into synaptic vesicles, from EC synaptic terminals using two photon microscopy in slices obtained from forebrain specific HCN1 deficient mice, global HCN1 knockouts and their wildtype littermates. This coupled with electrophysiology and pharmacology showed that HCN1 channels restrict the rate of exocytosis from a subset of cortical synaptic terminals within the EC and in this way, constrain non-action potential-dependent and action potential-dependent spontaneous release as well as synchronous, evoked release. Since HCN1 channels also affect post-synaptic potential kinetics and integration, our results indicate that there are diverse ways by which HCN1 channels influence synaptic strength and plasticity.

The hyperpolarization-activated cyclic nucleotide-gated (HCN) channels are voltage-gated ion channels that typically open at potentials more negative to −50 mV^1^,^2^,^3^,^4^. These channels are, thus, active at rest in most neurons. As the channels are permeable to Na^+^ and K^+^ ions, they generate a depolarizing current at rest and depolarize the resting membrane potential. In addition, HCN channels regulate the membrane resistance. Thus, these channels have a profound effect on intrinsic membrane excitability and synaptic potential integration, particularly in pyramidal cell dendrites where they are present in a high density^1^,^2^,^3^,^5^,^6^,^7^.

Interestingly, accumulating evidence from immunohistochemical studies indicates that HCN channels are also located pre-synaptically at select immature and mature synaptic terminals in the central nervous system^8^,^9^,^10^,^11^,^12^. Using electron microscopy coupled with immunogold labelling as well as electrophysiological recordings of miniature excitatory postsynaptic currents, we have previously shown that HCN channels present on select synaptic terminals targeting layer III pyramidal neurons in the mature entohrinal cortex (EC) are likely to restrict the release of glutamate by altering Ca^2+^ channel activity^9^. Thus, it can be hypothesised that alterations in pre-synaptic HCN channels at these synapses alter the rate of vesicle exocytosis. Additionally, since the EC receives inputs from cortical as well as subcortical regions^13^, it is not clear which neurons the synaptic terminals expressing HCN channels arise from.

In this study, we have addressed these questions by measuring the rate of release of FM1-43, a dye that is taken up into synaptic vesicles only, from EC synaptic terminals present in acute entorhinal-hippocampal slices obtained from adult mice in which HCN deletion was restricted to the forebrain only (HCN1^f/fcre^), HCN1 null mice in which HCN1 deletion was throughout the central nervous system (HCN1^−/−^) and their littermates. This together with electrophysiology and pharmacology strongly suggest that pre-synaptic HCN channels constrain non-action potential-dependent and action potential-dependent spontaneous release as well as evoked neurotransmitter release from select cortical synaptic terminals in the EC by restricting synaptic vesicle exocytosis. Our findings, therefore, suggest that that there are numerous and diverse cellular mechanisms by which HCN channels may modify neuronal and network excitability.

## Results

### Pre-synaptic HCN1 subunits located in cortical neurons regulate spontaneous excitatory synaptic release onto EC layer III pyramidal neurons

Four HCN subunits have thus far been cloned, of which HCN1 subunits are predominantly present in EC neurons, including EC layer III pyramidal neurons^14^,^15^. These subunits are expressed post-synaptically in these cells^14^ as well as on excitatory synaptic terminals that target these neurons^9^. Since we wished to study whether synaptic terminals in the EC expressing HCN1 subunits arose from cortical neurons, we decided to use HCN1^f/f,cre^ mice in which HCN1 subunit deletion was restricted to hippocampal and cortical principal (pyramidal and stellate) cells^16^,^17^. We verified this by performing immunogold immunohistochemistry on several brain regions using a selective HCN1 antibody^9^,^10^. In 540 sections obtained from 3 HCN1^f/f,cre^ mice and 3 wildtype littermates (referred to as HCN1^f/wt^), many HCN1 immunogold particles were located in dendrites in the EC and hippocampus of the HCN1^f/wt^ (Supp. Fig. 1a,b). Interestingly, HCN1 immunogold particles were present on asymmetric axon terminals in HCN1^f/wt^ EC too (Supp. Fig. 1a). The density of dendritic and synaptic HCN1 immunogold particles was substantially reduced in the HCN1^f/f,cre^ EC and hippocampus (Supp. Fig. 1a,b). In contrast, there were little differences in HCN1 expression between HCN1^f/f,cre^ and HCN1^f/wt^ in subcortical regions such as the lateral geniculate nucleus of the thalamus (Supp. Fig. 1a,b). Hence, HCN1 expression was selectively reduced in the cortex and hippocampus of the HCN1^f/f,cre^ mice.

**Figure 1:**
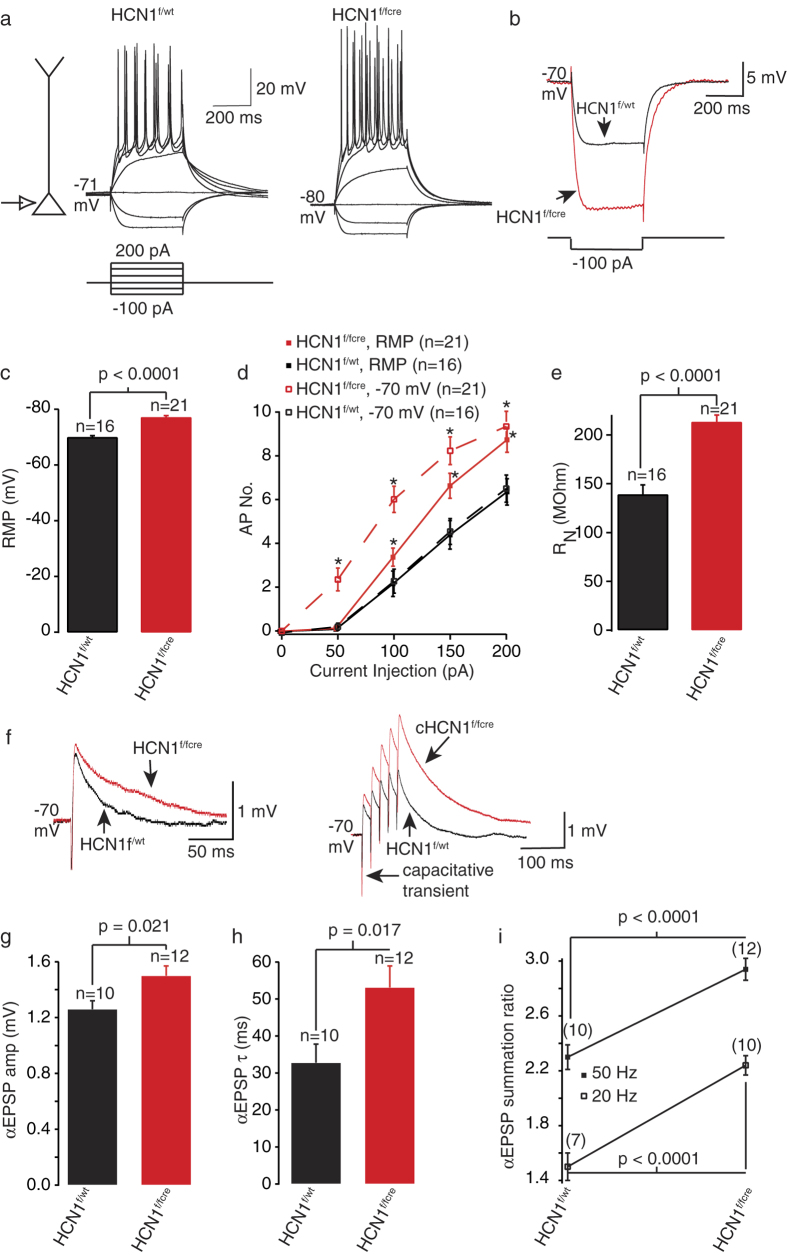
HCN1^f/fcre^ EC layer III neurons are hyperexcitable despite a hyperpolarized RMP. (**a**) Typical electrophysiological recordings obtained from HCN1^f/wt^ and HCN1^f/fcre^ EC layer III neuron somata at the normal RMP. The traces were obtained by applying a series of 400 ms hyperpolarizing and depolarizing current steps from −100 pA to + 200 pA as shown in the schematic. Note that the HCN1^f/fcre^ neuron RMP is substantially more hyperpolarized compared with that of the HCN1f^/wt^ neuron. The scale shown applies to both traces. (**b**) Representative traces obtained when 100 pA hyperpolarizing pulses were applied from a fixed potential of −70 mV to measure the R_N_. (**c–e**) Graphs depicting the mean RMP values, average numbers of action potentials elicited with 400 ms depolarizing current pulses applied either at the RMP (solid lines) or at −70 mV (dashed lines) and mean R_N_ measured at −70 mV using 100 pA hyperpolarizing current steps respectively. (**f**) Example single and 50 Hz trains of αEPSPs obtained by injecting alpha waveforms (see Methods) in HCN1^f/wt^ and HCN1^f/fcre^ neuron somata. The traces were obtained at a fixed potential of −70 mV. (**g,h**) Bar graphs showing the mean amplitude and decay time constant (τ) of single αEPSPs in HCN1^f/wt^ and HCN1^f/fcre^ neurons respectively. (**i**) The average summation ratios of 20 Hz (open squares) and 50 Hz (solid squares) trains of αEPSPs in HCN1^f/wt^ and HCN1^f/fcre^ neuron somata. The numbers of observations for each point are indicated in parenthesis. The significance (p) values are shown for all comparisons except for the graph in (**d**). Here, asterisks denote significance at p < 0.05 when compared with appropriate controls.

Whilst CA1 pyramidal neurons and EC layer II neurons have been characterized in the HCN1^f/fcre^ mice^15^,^16^, the properties of EC layer III neurons remain unexplored. Since both HCN1 and HCN2 are expressed in the EC^12^ and although it is expected that HCN1 predominantly contributes to the HCN channel current, I_h_, in EC layer III neurons^14^, it is necessary to determine whether I_h_ is ablated in these neurons in HCN1^f/fcre^ mice too. We, therefore, first made whole-cell current clamp recordings from mature HCN1^f/f,cre^ and HCN1^f/wt^ medial EC layer III pyramidal cell somata in the presence of glutamate and GABA receptor inhibitors (see Methods). HCN1^f/f,cre^ EC layer III somata had significantly negative resting membrane potentials (RMP) and greater input resistances (R_N_) than HCN1^f/wt^ neurons (Fig. 1a,b,c,e). As a consequence of the higher R_N_, considerably more action potentials were elicited with a given depolarizing step in HCN1^f/f,cre^ neurons than HCN1^f/wt^ at either the normal RMP or at a fixed potential of −70 mV (Fig. 1a,d). In addition, a single artificially generated excitatory post-synaptic potential (αEPSP; see Methods) waveform at a fixed membrane potential had substantially larger amplitudes and slower decay time constants in HCN1^f/f,cre^ neurons than HCN1^f/wt^ neurons (Fig. 1f–h). Thus, the summation of 20 Hz or 50 Hz trains of αEPSPs was markedly augmented in HCN1^f/f,cre^ neurons compared with HCN1^f/wt^ neurons (Fig. 1f,i).

External application of ZD7288 onto HCN1^f/wt^ EC layer III pyramidal neurons reduced the RMP, enhanced R_N_, augmented action potential firing, lengthened αEPSP decay time constants and boosted αEPSP summation (Supp. Fig. 2a–d). The effects of ZD7288 started to occur 5–10 min after application, with maximum effects observed within 15 min^14^,^18^. In contrast, treatment with ZD7288 for 15 min had little effect on HCN1^f/f,cre^ EC layer III neuron RMP, R_N_, action potential firing or αEPSP amplitude, kinetics or summation (Supp. Fig. 2). These findings confirm that, like in the HCN1 global knockout mouse (HCN1^−/−^) in which HCN1 expression is reduced throughout the brain^14^, HCN1 subunits also significantly contribute to I_h_ in HCN1^f/f,cre^ neurons. Moreover, these results are consistent with the lack of HCN1 immunogold labeling in HCN1^f/f,cre^ EC (Supp. Fig. 1).

**Figure 2:**
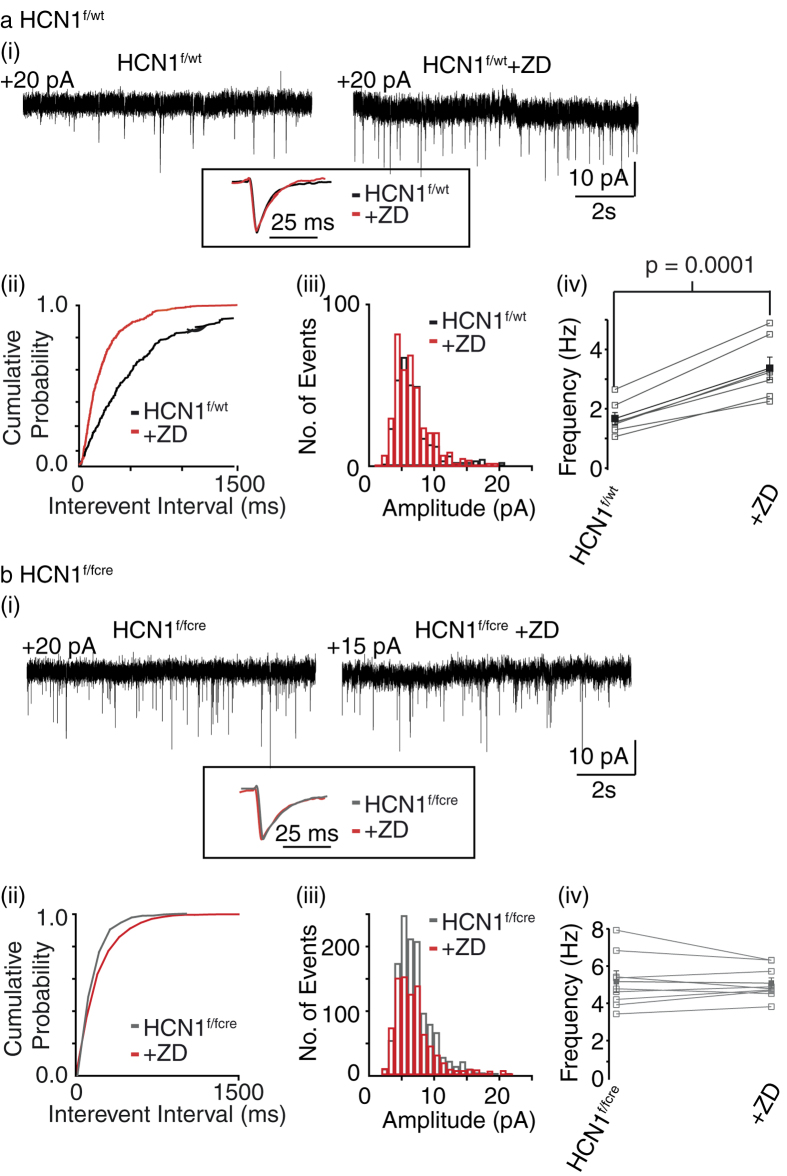
mEPSC frequency onto HCN1^f/fcre^ EC layer III neurons are enhanced compared with wildtypes. (**a**(i)**, b**(i)) Representative recordings of miniature EPSCs (mEPSCs) obtained from HCN1^f/wt^ and HCN1^f/fcre^ neurons before and following 15 min treatment with ZD7288 (ZD; 15 μM). The inset shows the average normalized EPSC obtained from the recordings. The outward holding current at −70 mV is indicated above each trace. The scales shown in each panel apply to both traces within the panel. (**a**(ii)**, a**(iii)**, b**(ii)**, b**(iii)) Cumulative probability curves and amplitude histograms associated with the traces shown in (**a**(i) and **b**(i)) respectively. (**a**(iv), **b**(iv)) Graph showing the individual (open squares) and mean (solid squares) frequency of mEPSCs recorded from 7 HCN1^f/wt^ and 9 HCN1^f/fcre^ neurons in the absence and presence of ZD7288.

All previous work involving understanding the role of pre-synaptic HCN1 subunits in neurotransmitter release have compared miniature excitatory post-synaptic current (mEPSC) frequency onto HCN1^−/−^ and wildtype EC layer III pyramidal neurons^9^. In the HCN1^−/−^ mice, though, HCN1 expression is abolished non-specifically throughout the nervous system^17^. Since the EC receives inputs from cortical regions and subcortical areas^13^ and because it is unknown whether cortical or subcortical afferents targeting EC layer III neurons may express HCN1 pre-synaptically, we initially investigated if mEPSC frequency was altered in the HCN1^f/f,cre^ slices compared with those from wildtype littermates. For these experiments, we obtained whole-cell voltage-clamp recordings from EC layer III pyramidal cell somata. To inhibit post-synaptic HCN channels, ZD7288 (15 μM) was included in the patch pipette solution (see Methods; 9,10. mEPSCs were measured in the presence of the Na^+^ channel inhibitor, tetrodotoxin and GABA receptor blockers^9^,^10^ (see Methods; Fig. 2). Under these conditions, the mEPSC frequency recorded from HCN1^f/f,cre^ neurons (5.17 ± 0.5 Hz, n = 9) was substantially greater than that obtained from HCN1^f/wt^ neurons (1.67 ± 0.2, n = 7, p < 0.0001; Fig. 2a,b). The amplitudes and kinetics of the mEPSCs, though, were similar (Supp. Table 1). Additional external application of ZD7288 (15 μM) for 15 min significantly enhanced mEPSC frequency by 103.5 ± 7.7% (n = 7, p = 0.0001) onto HCN1^f/wt^ neurons but not HCN1^f/f,cre^ neurons (% increase in mESPC frequency in HCN1^f/fcre^ with ZD7288 = 1.1 ± 4.3%, n = 9, p = 0.75; Fig. 2a,b). Again mEPSC amplitudes or shapes were not affected (Supp. Table 1). Since hippocampal afferents are not known to target EC layer III pyramidal neurons^13^ and because subcortical HCN1 subunit expression in HCN1^f/f,cre^ mice is unchanged (Supp. Fig. 1), these results indicate that cortical, but not subcortical, neurons synapsing onto EC layer III pyramidal neurons are likely to express pre-synaptic HCN1 subunits. Further work beyond the scope of the present study would be required to determine which cortical neurons express HCN1 subunits in their synaptic terminals.

### HCN1 subunits in EC layer III cortical synapses influence exocytosis

**Figure 3:**
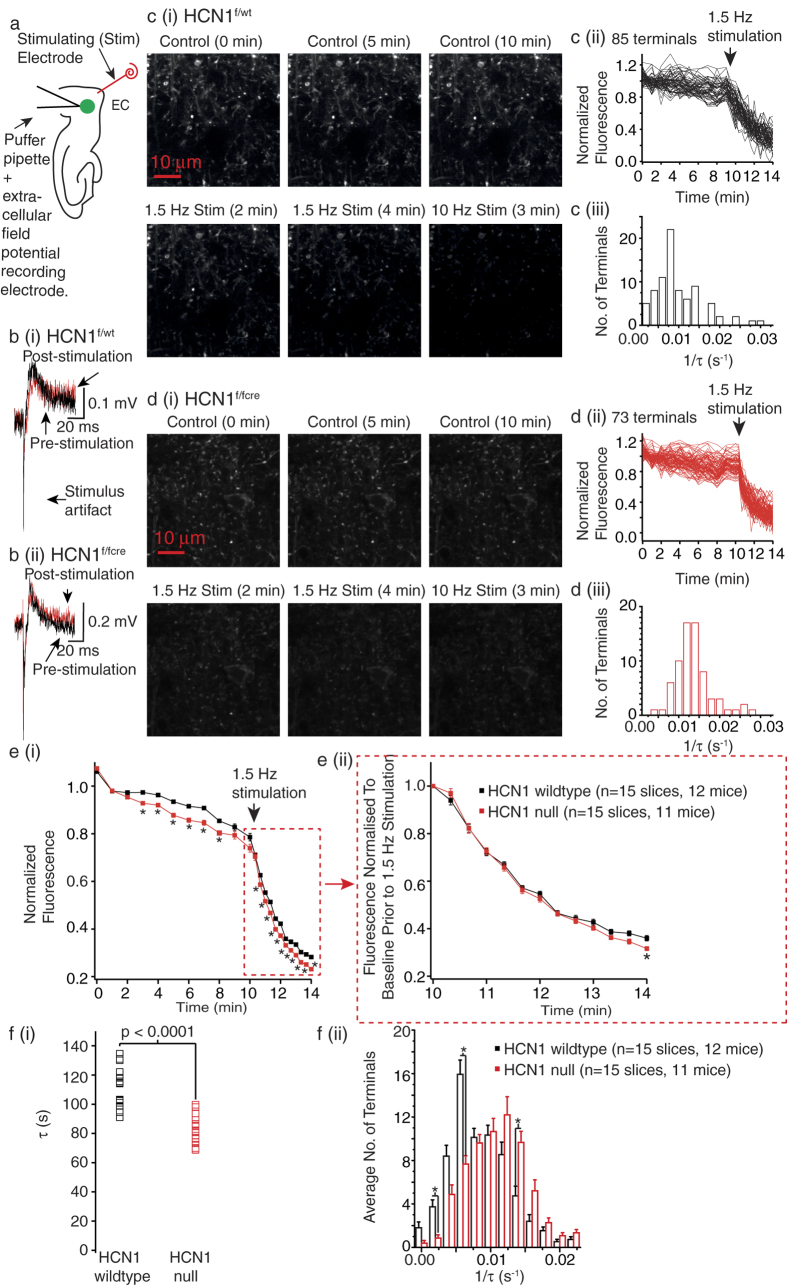
HCN1 deletion enhances synaptic vesicle exocytosis. **a** Schematic showing that FM1-43 was locally applied within EC layer III. (**b**) Superimposed example field EPSPs recorded in HCN1 wildtype (**b**(i)) and HCN1 null (**b**(ii)) slices during control conditions and following FM1-43 unloading after 10 Hz stimulation. (**c**(i)**, d**(i)) Representative images of terminals loaded with FM1-43 in HCN1 wildtype and HCN1null slices respectively. The scale shown in each panel applies to all images within the panel. (**c**(ii)**, d**(ii)) Quantification of the FM1-43 fluorescence of all individual terminals from the example HCN1 wildtype slice and HCN1 null slices shown in (**c**(i) and **d**(i)) respectively (see Methods for details). (**c**(iii)**, d**(iii)) Histograms showing the de-staining rates obtained by fitting the time-course of the fluorescence changes in terminals during 1.5 Hz stimulation shown in (**c**(ii) and **d**(ii)) respectively. (**e**(i)) Graph depicting the average normalised FM1-43 fluorescence in 15 HCN1 wildtype and 15 HCN1 null slices. (**e**(ii)) shows the data obtained following 1.5 Hz stimulation if the active FM1-43 fluorescence is normalized to the baseline prior to the 1.5 Hz stimulation. For data shown in (**e**(i) and **e**(ii)) significance was determined using a two-way ANOVA followed by unpaired t-tests with the p value adjusted for multiple comparisons using the Bonferroni-Dunn Method (Supp. Table 3). The decay rates of changes in fluorescence with time following 1.5 Hz stimulation were also measured. The median decay time constants (τ) obtained from 15 HCN1 wildtype and 15 HCN1 null slices are displayed in (**f**(i)). (**f**(ii)) A histogram to show the distribution of the τ during the 1.5 Hz stimulation for terminals present in 15 HCN1 wildtype and 15 HCN1 null slices. Two-tailed Mann Whitney U tests with the p value adjusted for multiple comparisons using a Bonferroni Constant were used to determine significance (Supp. Table 4). Significance levels at p < 0.05 are indicated using an asterisk.

Next, we tested whether pre-synaptic HCN1 channels affect the rate of exocytosis from HCN1 null synaptic terminals located in medial EC layer III present in brain slices. For this, we loaded synaptic vesicles in medial EC layer III with a fluorescent dye, FM1-43. FM1-43 partitions into cell membranes and is, thus, incorporated into synaptic vesicles during endocytosis and released upon exocytosis^19^,^20^. We locally applied the FM1-43 dye into HCN1^f/f,cre^, HCN1^−/−^, HCN1^f/wt^ and HCN1^+/+^ EC layer III whilst stimulating afferents extracellularly at 10 Hz for 3 min to induce synaptic vesicle exocytosis and dye uptake via subsequent endocytosis (as described previously^21^,^22^,^23^, Fig. 3a). The stimulation strength was adjusted to obtain single field EPSPs with a mean amplitude of 0.2 mV (Fig. 3b, Supp. Table 2). Following FM1-43 loading, ADVASEP-7, a dye that quenches FM1-43 but is lipid insoluble, was washed in to remove any remaining FM1-43 in the extracellular space. This allows visualization of individual fluorescent puncta with two-photon microscopy (Fig. 3c,d^21^,^22^,^23^,^24^). Between 50 to 100 putative synaptic terminals per slice could be identified. These were considered to be synaptic terminals if, in the presence of ADVASEP-7, further extracellular stimulation, initially at 1.5 Hz for 4 min followed by 10 Hz stimulation for 3 min to induce maximal exocytosis, resulted in a greater than 40% loss of fluorescence from the puncta (Fig. 3c,d; see Methods). Consistent with previous studies^21^, this stimulation paradigm did not induce plasticity as the field EPSP amplitude and decay time constants before and after extracellular stimulation in HCN1^f/wt^, HCN1^+/+^, HCN1^f/fcre^ and HCN1^−/−^ slices were very similar (Fig. 3b, Supp. Table 2). Moreover, since there were little differences in alterations in puncta fluorescence between HCN1^f/fcre^ and HCN1^−/−^ slices or HCN1^f/wt^ and HCN1^+/+^ slices, we pooled data from HCN1^f/fcre^ and HCN1^−/−^ slices (HCN1 null data) and wildtype slices (HCN1^f/wt^ and HCN1^+/+^).

Interestingly, there was an overall increase in the time-dependent fluorescence changes in puncta in HCN1 null slices (n = 15, 11 mice) compared with wildtype slices (n = 15, 12 mice) under baseline conditions as well as during the 1.5 Hz stimulation (Fig. 3e(i); p < 0.0001, F(22,322) Supp. Table 3). Additional statistical analysis showed that there were significant differences in fluorescence between HCN1 null slices and wildtype slices at the majority of time points (Fig. 3e(i), Supp. Table 3). Since these experiments were done in the absence of sodium channel inhibitors, these results indicate enhanced exocytosis during action potential-dependent and non-action potential-dependent spontaneous release from HCN1 null boutons compared with wildtype boutons. Moreover, because there were significant changes in puncta fluorescence between HCN1 null slices and wildtype slices during the 1.5 Hz stimulation, this suggests that pre-synaptic HCN1 channels restrict exocytosis during synchronous (evoked) release too.

Because the baseline before stimulation differed significantly between HCN1 null and wildtype slices (Fig. 3e(i)), we further examined the effects of HCN1 channels on evoked release by normalizing the changes in fluorescence during the 1.5 Hz stimulation to the baseline values just prior to the stimulation (Fig. 3e(ii)). With this, the time-dependent changes in fluorescence were significantly different in HCN1 null slices (n = 15, 11 mice) compared with wildtype slices (n = 15, 12 mice; p = 0.0198, F(12,364), Supp. Table 3). In addition, the average remaining puncta fluorescence following 4 min of 1.5 Hz stimulation in HCN1 null slices (31.6 ± 0.8%, n = 15 slices (1062 terminals), 11 mice) was significantly less than in HCN1 wildtype slices (36.0 ± 1.1%, n = 15 slices (1066 terminals), 12 mice; p = 0.0189, Supp. Table 3). Hence, these findings further support the notion that HCN1 channels restrict exocytosis during evoked release.

As has been described previously by Ahmed and Siegelbaum^21^, we also determine the rate of FM1-43 dye release by fitting the time course of the change in each fluorescence in individual puncta following 1.5 Hz stimulation (see Methods). The median (middle) rates of dye release in 15 HCN1 null slices during the 1.5 Hz stimulation were significantly greater than that from 15 HCN1 wildtype slices (Fig. 3f(i)). However, since HCN1 is expressed only in approximately one third of synaptic boutons in the EC^9^, there was, as expected, a great variation in the rate of release between individual terminals in both HCN1 null and wildtype slices. We, therefore, determined whether there were differences in populations of synaptic terminals with particular decay time constant ranges within HCN1 null and wildtype slices (Fig. 3f(ii), Supp. Table 4). In HCN1 wildtype slices, there were 35% more synaptic terminals in which dye release occurred between 0.006 s^−1^ and 0.008 s^−1^ than in HCN1 null slices (Fig. 3f(ii)). In contrast, in HCN1 null slices there were 20.22% more synaptic terminals in which the de-staining rate was between 0.014 s^−1^ and 0.016 s^−1^ compared with HCN1 wildtype slices (Fig. 3f(ii)). Altogether, these findings strongly suggest that HCN1 channels present in a subset of mature EC synaptic boutons reduce the rate of synaptic vesicle exocytosis during evoked release.

**Figure 4:**
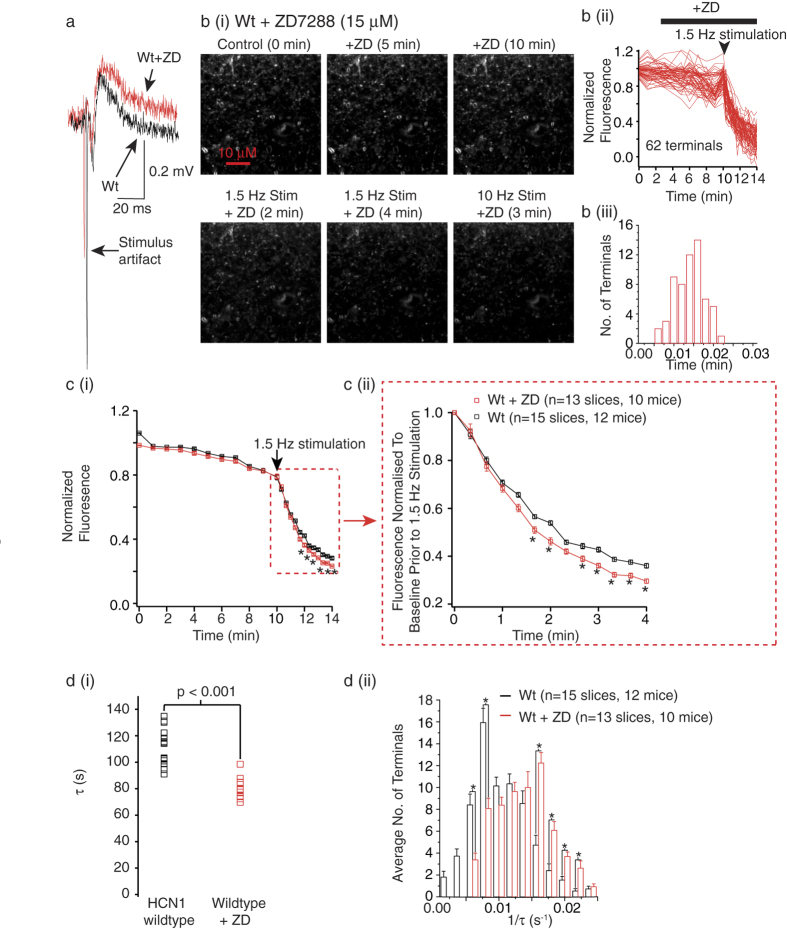
Pharmacological inhibition of HCN1 channels increases synaptic vesicle exocytosis. (**a**) Field EPSPs under control conditions and following 20 min treatment of ZD7288 recorded in EC layer III. (**b**(i)) Images of FM1-43 loaded terminals in a wildtype (Wt) slice before and during treatment with ZD7288 in the absence and following 1.5 Hz and 10 Hz extracellular stimulation. The scale shown applies to all images within the panel. Quantification of the FM1-43 fluorescence and dye release rates from all individual terminals from this slice are shown in (**b**(ii) and **b**(iii)) respectively. (**c**(i)) Graph illustrating the changes in FM1-43 fluorescence in all wildtype slices in the absence and presence of ZD7288. The graph shows the changes in active FM1-43 fluorescence if normalized to the average of the first three points. (**c**(ii)) represents the same data when normalized to the FM1-43 fluorescence values just prior to the 1.5 Hz stimulation. (**d**(i)) Median decay time constant values from 15 wildtype slices and 13 wildtype slices treated with ZD7288. The average de-staining rates of the terminals during 1.5 Hz stimulation in slices treated with ZD7288 and untreated slices. Asterisks indicate significance at p < 0.05 (Supp. Tables 3 and 4).

Next we tested whether the pharmacological inhibitor, ZD7288 (15 μM) would alter synaptic vesicle exocytosis in HCN1 wildtype slices. For these experiments, we first obtained field EPSPs and images of putative wildtype EC layer III boutons loaded with FM1-43 in the absence of ZD7288. We subsequently applied ZD7288 to the slice and acquired images for a further 10 min without extracellular stimulation. This was followed by extracellular stimulation of afferents for 4 min at 1.5 Hz and then 3 min at10 Hz (Fig. 4). The protocol was designed in this manner as 15 μM ZD7288 is not likely to have non-specific effects on synaptic transmission during a 20 min time period^9^,^25^. Field EPSPs were also obtained at the end of the recording and, as expected with HCN channel block (see above), the amplitude and decay of the EPSPs were significantly enhanced in the presence of ZD7288 compared with that in the absence (Fig. 4a; Supp. Table 2). Since ZD7288 takes 5–10 min to begin to inhibit HCN channels (see above)^18^, the baseline fluorescence for the first 10 min whilst ZD7288 was being applied was similar to that of wildtypes (Figs. 4b,c(i), Supp. Table 3). With 4 min extracellular stimulation at 1.5 Hz, though, there was a considerable decrease in fluorescence from synaptic terminals present in wildtype slices treated with ZD7288 than that in untreated slices (Fig. 4c(i)). Indeed, a comparison of the time-dependent changes in fluorescence in wildtype slices treated with ZD7288 with untreated slices showed that inhibition of HCN channels substantially enhanced FM1-43 dye release (p < 0.0001, F(22,598), Supp. Table 3). To further examine the changes occurring during evoked release per se, we again normalized the puncta fluorescence during the 1.5 Hz stimulation to the baseline prior to 1.5 Hz stimulation (Fig. 4c(ii)). This further supported the notion that inhibition of HCN channels significantly enhances FM1-43 dye release during evoked release (p = 0.0059, F(12,338), Supp. Table 3). Indeed, the change in remaining puncta fluorescence after 4 min of 1.5 Hz stimulation in the absence and presence of ZD7288 were 36.0 ± 1.0% (n = 15 slices (1066 terminals), 12 mice) and 29.6 ± 1.0% (n = 13 slices (891 terminals), 10 mice) respectively (p = 0.0008; Fig. 4c(ii), Supp. Table 3).

We also investigated if the rate of de-staining of individual puncta during the 1.5 Hz stimulation paradigm differed between slices treated with ZD7288 and untreated slices. The rate of dye release in 13 wildtype slices in the presence of ZD7288 was significantly greater than in 15 untreated wildtype slices (Fig. 4d(i)). Interestingly, the distribution of the destaining rates of individual synaptic terminals in the presence of ZD7288 was varied, with 25.7% of terminals exhibiting rate constants between 0.014 s^−1^ to 0.016 s^−1^ more than untreated wildtype terminals (Fig. 4d(ii), Supp. Table 4). The changes in the rate of de-staining in the presence of ZD7288 were comparable to those observed in HCN1 null slices (Fig. 3, Fig. 4). Thus, these findings further strongly re-inforce the notion that native HCN1 subunits located in a subset of synaptic terminals within EC layer III significantly reduce the rate of exocytosis during synchronous release.

## Discussion

In this study, using HCN1 null mice and their respective wildtype littermates together with FM1-43 imaging, electrophysiology, two photon microscopy and pharmacology, we show that endogenous HCN1 channels present in a subset of adult EC synaptic terminals substantially restrict the rate of exocytosis and thereby, spontaneous non-action potential- and action potential- dependent as well as synchronous (evoked) synaptic transmission (Figs 2, 3, 4). These are likely to be synaptic terminals belonging to cortical neurons as the changes in synaptic transmission were similar in mice in which HCN1 deletion was restricted to the forebrain only (HCN1^f/f,cre^) and HCN1 global knockout mice (HCN1^−/−^; Fig. 2). These findings build on our previous studies^9^,^10^ and provide direct evidence that native HCN1 channels located on mature cortical synaptic terminals restrict synaptic release and modify different modes of neurotransmitter release. This, together with their effects on intrinsic excitability (Figs 2 and 3), indicate that HCN1 channels shape EC neuronal excitability in numerous and diverse ways.

This, together with their effects on intrinsic excitability (Fig. 1, Supp. Fig. 2), indicate that HCN1 channels shape EC neuronal excitability in numerous and diverse ways.

In the EC, the majority of HCN1 subunits are located on dendrites^9^. However, there is a small (approx. 30%) of assymetric (presumably excitatory) and symmetric synaptic terminals that also express HCN1 subunits (Supp. Fig. 19). Indeed, in this study we estimate that there is between 20% and 35% of terminals that are likely to have a greater rate of exocytosis in the absence of HCN channels than wildtypes (Figs 3 and 4). Interestingly, HCN1 channels do not affect spontaneous inhibitory synaptic release in the EC^9^. HCN1 channels, however, are present in inhibitory synaptic terminals in many brain regions where they may modify neurotransmitter release^8^,^11^,^26^,^27^. Moreover, HCN1 channels present in immature excitatory synaptic terminals in the hippocampus and Calyx of Held are also likely to modulate neurotransmission^28^,^29^. Thus, it remains a possibility that in these inhibitory and immature excitatory synapses, pre-synaptic HCN1 channels may also modify synaptic vesicle exocytosis and thereby influence synaptic release. Indeed, altered synaptic vesicle exocytosis has been implicated in cultured rat lactotrophs in which overexpression of HCN2 channels results in cAMP mediated enhanced exocytosis as measured by changes in capacitative transients from the terminals^30^. Remarkably, HCN1 channels may affect neurotransmitter uptake into synaptic vesicles in some synapses too ref. 29. Moreover, whilst our study has focused on the rate of exocytosis, it is possible that HCN channels present on synaptic boutons may alter endocytosis too, alterations in which cannot be easily measured using FM1-43 in the acute brain slice preparation. If so, there may be diverse molecular mechanisms by which pre-synaptic HCN1 channels may regulate neurotransmitter release.

How might HCN1 channels affect synaptic vesicle exocytosis in mature EC excitatory synapses? A reduction in HCN1 channel function significantly hyperpolarizes the RMP and simultaneously increases the membrane resistance of cortical pyramidal neurons (Fig. 1, Supp. Fig. 2). Moreover, in the EC, pre-synaptic HCN1 channels present in excitatory synapses are co-localised with T-type Ca^2+^ channels^9^. The decrease in pre-synaptic HCN1 activity in these synapses enhances T-type Ca^2+^ channel function^9^. Thus, the resting Ca^2+^ concentration within these synapses may be greater in the absence of HCN1 channels. This may be one reason for the increased spontaneous release in HCN1 null synapses compared with wildtypes (Fig. 3). Further, by increasing the membrane input resistance (Fig. 1, Supp. Fig. 2), the lack of pre-synaptic HCN1 channels may alter the activity of voltage-gated Ca^2+^ channels too. In this manner, HCN1 channels might then constrain synchronous release too.

Neurotransmitter release from synapses is the predominant mechanism for information transfer between neurons^19^,^31^,^32^. By having a significant impact on synaptic release from synapses targeting EC layer III neurons, pre-synaptic HCN1 channels are likely to play a significant role in modulating synaptic strength and synaptic plasticity and thereby information storage in these neurons. The axons of EC layer III neurons project to the hippocampus^33^ and alterations in their activity and output have been suggested to impact spatial navigation^34^,^35^ and memory formation^36^,^37^. Thus, the dynamic tuning of neurotransmitter release onto EC layer III neurons by HCN1 channels could play a pivotal role in impacting these physiological processes.

## Methods and Materials

### Ethical Approval

All procedures were approved by the UCL Animal Welfare and Ethics Board and the UK Home Office and were performed in accordance with the relevant guidelines and regulations of the UK Home Office Animals (Scientific Procedures) Act 1986 and specifications on the Home Office Project license held by the senior author.

### HCN1^f/fcre^ and HCN1^−/−^ Mice

The HCN1^f/fcre^ mice were a kind gift from Prof. M. F. Nolan (Edinburgh University, UK). HCN1^f/wt^ (mice containing the floxed HCN1 allele only) were crossed with mice expressing Cre only to obtain HCN1^f/fcre^ mice and wildtype littermates (HCN1^f/wt^; as described previously^16^,^17^) and maintained on a 129SVEV/C57 background. HCN1^−/−^ mice and their respective wildtypes (HCN1^+/+^) were obtained as previously described and maintained on 129SVEV background^14^. The mice used in this study were bred at UCL School of Pharmacy under a 12 hr light/dark cycle and had unlimited access to food and water. All mice had been backcrossed for more than 10 generations. 6–9 week old mice of either sex were used for this study. The experimenter was blind to the genotype of the mouse.

### Electrophysiology experiments

Entorhinal-hippocampal slices were obtained as previously described^38^. Animals were terminally anaesthetized using a mixture of ketamine/xylazine solution (K113; Sigma, UK). Once fully anesthetized, the mice were intra-cardially perfused with an ice-cold cutting solution of the following composition (mM): 110 choline chloride, 2.5 KCl, 1.25 NaH_2_PO_4_, 25 NaHCO_3_, 0.5 CaCl_2_, 7 MgCl_2_, 7 glucose, bubbled with 95% O_2_/5% CO_2_ (pH 7.2). Mice were subsequently decapitated and the brain removed and placed in ice-cold cutting solution. 350 μm thick slices were obtained using a Leica VT1200S (Leica, UK) and placed in a recovery chamber containing external solution (mM): 125 NaCl, 2.5 KCl, 1.25 NaH_2_PO_4_, 25 NaHCO_3_, 2 CaCl_2_, 2 MgCl_2_, 10 glucose, bubbled with 95% O_2_/5% CO_2_ (pH 7.2). Slices were stored in this chamber at room temperature. For recording purposes, slices were placed in a chamber containing the external solution maintained at 32–36 °C and viewed using an Olympus BX51W1 equipped with differential infra-red optics. The slices were perfused with external solution. The external solution for whole-cell current clamp recordings was supplemented with 0.05 mM DL-AP5, 0.01 mM CNQX, 0.01 mM bicuculline and 0.001 mM CGP 55845. For mEPSC recordings, the external solution contained 0.001 mM tetrodotoxin, 0.01 mM bicuculline and 0.001 mM CGP 55845. The internal recording pipette solution for whole-cell current clamp was composed of (in mM): 120 KMeSO_4_, 20 KCl, 10 HEPES, 2 MgCl_2_, 0.2 EGTA, 4 Na_2_-ATP, 0.3 Tris-GTP, 14 Tris-phosphocreatine; pH was adjusted to 7.3 with KOH. For mEPSC recordings, this internal solution was supplemented with 0.015 mM ZD7288. Pipettes containing these internal solutions had resistances of 5–12 MΩ. Whole-cell current clamp recordings were obtained using an AxoClamp 2B amplifier under bridge-mode (Molecular Devices, UK), filtered at 10 kHz and sampled at 50 kHz. mEPSC recordings were obtained using a Axopatch 200B amplifier (Molecular Devices, UK), filtered at 1 kHz and sampled at 10 kHz. Series resistance was usually in the order of 10–30 MΩ and was approx. 70% compensated for the whole-cell voltage clamp recordings. Recordings were discarded if the series resistance increased by more than 20% during the course of the recordings. Data were acquired using pClamp 10.4 (Molecular Devices, UK).

400 ms square hyperpolarizing and depolarizing current injections were applied to measure R_N_ and generate action potentials. Articifial EPSPs, αEPSPs, were generated by current injection of the order:


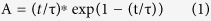


where A is the amplitude of the current injected and τ is the rise time constant.

All reagents for the internal and external solutions were obtained from Sigma-Aldrich UK. DL-AP5, CNQX, bicuculline, CGP 55845 and ZD7288 were obtained from Abcam UK.

#### Data Analysis for electrophysiology experiments

Clampfit (v10.4) was used for analysis of whole-cell current clamp recordings. To calculate input resistance (R_N_), the difference in steady-state voltage in the last 25 ms elicited by a 100 pA, 400 ms hyperpolarizing step at −70 mV was divided by the applied current. Action potentials elicited by 400 ms depolarizing steps were counted. The αEPSP decay time constants were obtained by fitting the double exponential function:


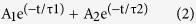


where τ1 and τ2 represent time constants of the initial and falling phase of the αEPSPs. Since τ2 is most affected by the changes in the membrane time constant that are caused by I_h_, we only compared τ2 values in the absence and presence of HCN channels. The summation ratio of αEPSPs was calculated as the ratio of the peak of the 5^th^ αEPSP to that of the 1^st^ αEPSP.

mEPSC recordings were analysed using Mini-analysis program (v6.07, Synpatosoft, USA). Events >1.5 pA in amplitude (i.e. all events above baseline noise level) and with rise times of <2 ms were detected and used for analysis. Decay times and amplitudes of these events were obtained by fitting the averaged EPSC or IPSC with a single exponential equation:


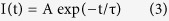


where I is the current amplitude at any given time (t), A is the peak amplitude of the EPSC or IPSC and τ is the decay time constant.

Unpaired t-tests were employed to determine significance in resting membrane potential (RMP) values, R_N_ values, numbers of action potentials at a given current injection, αEPSP amplitudes and decay time constants as well as mEPSC frequency, amplitude and decay time constants between HCN1^f/wt^ and HCN1^f/fcre^ neurons. Paired t-tests were used to assess the effects of ZD7288 on these parameters in HCN1^f/wt^ and HCN1^f/fcre^ neurons.

### FM1-43 imaging experiments

Slices from HCN1^f/fcre^, HCN1^f/wt^, HCN1^−/−^ and HCN1^+/+^ of either sex were prepared as described above and placed in a chamber on a Prairie Ultima two-photon system. The slices were perfused with the external solution described above and supplemented with 0.05 mM DL-AP5, 0.01 mM bicuculline and 0.001 mM CGP 55845. The FM1-43 loading and unloading protocol utilised was similar to that described by Ahmed and Siegelbaum^21^. Briefly, a patch pipette (3–5 MOhms) containing 125 μM FM1-43 (Cambridge Biosciences, UK) was placed in EC layer III. A stimulation electrode was placed in EC layer I. Once stable field EPSPs could be recorded, positive pressure was applied for 5 min to release the FM1-43. Simultaneously, EC layer I was stimulated at 10 Hz for 3 min. The patch pipette containing the FM1-43 dye was then replaced with one containing the external solution only and the slice perfused with 200 μM ADVASEP-7 (Cambridge Biosciences, UK) for 30–40 min to remove any extracellular dye bound to the tissue. Single puncta labelled with FM1-43 could then be visualised using the Prairie Ultima two-photon system. An area of 30 μm × 30 μm was selected and a z-stack of 10 images, 1 μm were taken. A series of images were initially obtained every minute over a period of 10–20 min (baseline recording). To ascertain FM1-43 destaining rates, the afferents in EC layer I was stimulated at 1.5 Hz for 4 min and images were obtained every 20 s during this time period. The afferents were then stimulated at 10 Hz for 3 min to maximally unload the dye. This allowed us to determine the background fluorescence and normalize the fluorescence. Only those terminals that showed a greater than 40% loss of fluorescence upon electrical stimulation were considered for analysis.

#### Data analysis of FM1-43 imaging data

All data-analysis was performed using MATLAB. Consistent with criteria used by Ahmed and Siegelbaum^21^, roughly circular puncta that had a fluorescence intensity greater than 1.5 × standard deviation of the background fluorescence and a diameter between 0.3 μm and 3 μm were selected for analysis. The average fluorescence for each identified puntum was measured. Indivdual punta were tracked over time during baseline recordings and following stimulation. Slices in which puncta underwent significant lateral movement (greater than 2 μm) or more than 30% loss of fluorescence occurred without stimulation were discarded. The active component of the punta was calculated by subtracting background fluorescence from the residual fluorescence at a given time point. The active component was then normalized to the average of the maximum three values acquired at the start of the experiment (referred to as ‘normalised fluorescence’; Figs 3 and 4). The changes in fluorescence for each punta with time were calculated in each slice and the average changes in fluorescence with time for each slice were obtained. The time constant for each fluorescence intensity decay (τ) was calculated by fitting a single exponential decay function to the fluorescence intensity curve during the 1.5 Hz stimulation unloading period. The fit was constrained to the first time point following the 1.5 Hz stimulation.

All statistical analysis were carried out using GraphPad Prism 7.01. The average changes in FM1-43 de-staining during baseline and following 1.5 Hz stimulation between individual wildtype and HCN1 null slices were determined to be normally distributed and therefore, tested for significance using a two-way ANOVA followed by unpaired t-tests with the p value adjusted for multiple comparisons using the Bonferroni-Dunn Method. Since the decay time constants were determined to be not normally distributed, two-tailed Mann-Whitney U tests with the p value adjusted for multiple comparisons using a Bonferroni Constant were used to determine significant differences between decay time constants in the absence and presence of HCN channels. All data are presented as means ± standard errors.

### HCN1 immunoreactivity experiments

Three HCN1^f/fcre^ and HCN1^f/wt^ mice of either sex were deeply anaesthetized by intraperitoneal injection of ketamine-xylazine and transcardially-perfused with ice-cold fixative containing 4% paraformaldehyde, 0.05% glutaraldehyde, and 15% saturated picric acid in 0.1 M phosphate buffer (PB, pH 7.4). After perfusion, brains were removed and immersed in the fixative for 2 hr. Tissue blocks were washed in 0.1 M PB. 60 μm coronal sections were cut with a vibratome (Leica V1000), as described previously^39^. Free-floating sections were incubated in 10% NGS diluted in TBS for 1 hr at room temperature. Sections were then incubated for 48 h in anti-HCN1 antibodies (Neuromab) at a final protein concentration of 1–2 μg/ml diluted in TBS containing 1% NGS. After several washes in TBS, sections were incubated for 3 hr in goat anti-mouse IgG coupled to 1.4 nm gold (Nanoprobes Inc., Stony Brook, NY) diluted 1:100 in TBS containing 1% NGS. After several washes in phosphate-buffered saline (PBS), the sections were postfixed in 1% glutaraldehyde diluted in the same buffer for 10 min. They were washed in double distilled water, followed by silver enhancement of the gold particles with a HQ Silver kit (Nanoprobes Inc., Stony Brook, NY). Then, sections were treated with osmium tetraoxide (1% in 0.1 M PB), block-stained with uranyl acetate, dehydrated in graded series of ethanol and flat-embedded on glass slides in Durcupan (Fluka) resin. Regions of interest were cut at 70–90 nm on an ultramicrotome (Reichert Ultracut E, Leica, Austria) and collected on 200-mesh nickel grids. Staining was performed on drops of 1% aqueous uranyl acetate followed by Reynolds’s lead citrate. Ultrastructural analyses were performed in a Jeol-1010 electron microscope (Japan). A monoclonal antibody against HCN1 (Clone N70/28) was obtained from the NeuroMab Facility (University of California, Davis, USA). No labelling was observed if the primary antibody was either omitted or replaced with 5% (v/v) normal serum.

#### HCN1 immunogold quantification

To establish the relative abundance of HCN1 quantification of immunolabeling was performed from 60 um coronal slices as described^39^. For each of three HCN1^f/fcre^ and HCN1^f/wt^ animals, three samples of tissue were obtained (nine total blocks for each genotype and brain region). Electron microscopic serial ultrathin sections were cut close to the surface of each block because immunoreactivity decreased with depth. Randomly selected areas were captured at a final magnification of 45,000X, and measurements covered a total section area of around 5000 μm^2^. Dendritic shafts, dendritic spines and axon terminals were assessed for the presence of immunoparticles. The density of HCN1 immunoparticles, measured as immunoparticles/μm^2^, was calculated in each brain region. Unpaired t-tests were utilized to compare the numbers of immunogold particles in HCN1^f/wt^ and HCN1^f/fcre^ tissue.

## Additional Information

**How to cite this article**: Huang, Z. *et al*. HCN1 channels reduce the rate of exocytosis from a subset of cortical synaptic terminals. *Sci. Rep.* **7**, 40257; doi: 10.1038/srep40257 (2017).

**Publisher's note:** Springer Nature remains neutral with regard to jurisdictional claims in published maps and institutional affiliations.

## Supplementary Material

Supplementary Information
