# Supplementary material for: HCN1 channels reduce the rate of exocytosis from a subset of cortical synaptic terminals
Source: Sci Rep. 2017 Jan 10;7:40257. doi: 10.1038/srep40257 (PMC5223132; doi:10.1038/srep40257)
Supplement: Supplementary Information [file srep40257-s1.pdf]

HCN1 channels reduce the rate of exocytosis from a subset of cortical synaptic terminals.

Abbreviated Title: Pre-synaptic HCN1 channels and exocytosis

Zhuo Huang[1], Gengyu Li[1], Carolina Aguado[2], Rafael Lujan[2] and Mala M. Shah[1*].

[1]UCL School of Pharmacy, University College London, London, WC1N 1AX, UK;

[2]Departamento de Ciencias Medicas, Universidad de Castilla-La Mancha, 02006 Albacete, Spain

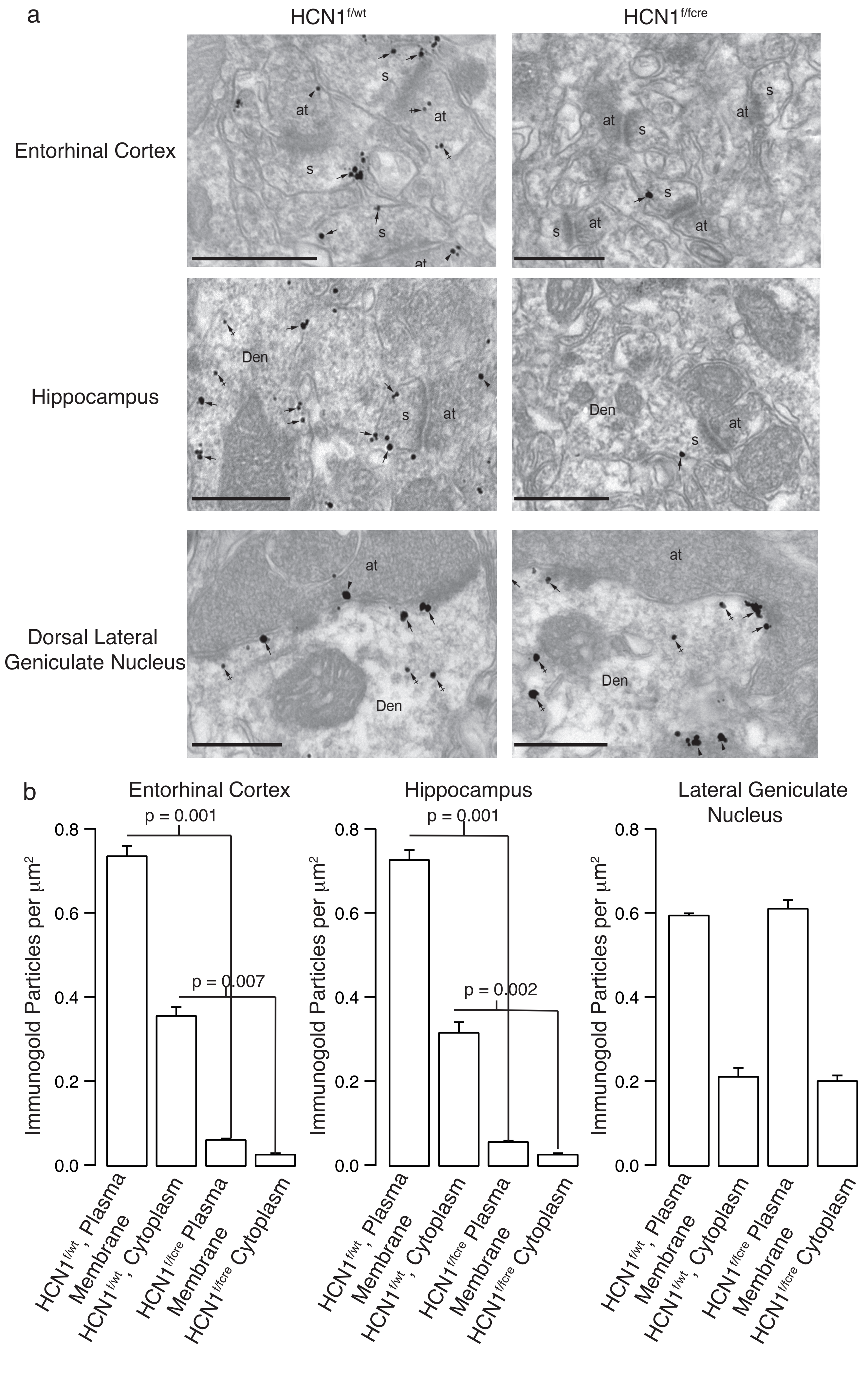
a
HCN1 f/wt
HCN1 f/fcre
Entorhinal Cortex
Hippocampus
Dorsal Lateral Geniculate Nucleus
s
at
Den
b
Entorhinal Cortex
Hippocampus
Lateral Geniculate Nucleus
p = 0.001
p = 0.007
p = 0.002
Immunogold Particles per μm2
0.0
0.2
0.4
0.6
0.8
HCN1 f/wt, Plasma Membrane
HCN1 f/wt, Cytoplasm
HCN1 f/fcre Plasma Membrane
HCN1 f/fcre Cytoplasm

Supp Fig 1: HCN1 expression is reduced significantly in entorhinal cortex (EC) and hippocampus. a Example electron micrographs showing expression of HCN1 immunogold particles in $HCN1^{f/wt}$ and $HCN1^{f/fcre}$ tissue sections obtained from the EC, hippocampus and dorsal lateral geniculate neurons. Arrows indicate HCN1 immunogold particles present in axon terminals (at), dendrites (den) and spines (s). Each scale bar represents 500 nm. b Quantification of HCN1 immunoreactivity associated with plasma membranes or cytoplasm in $HCN1^{f/wt}$ and $HCN1^{f/fcre}$ EC, hippocampus and lateral geniculate nucleus. Where appropriate, significance levels (p values) have been indicated.

Huang et al., Supp Fig 2

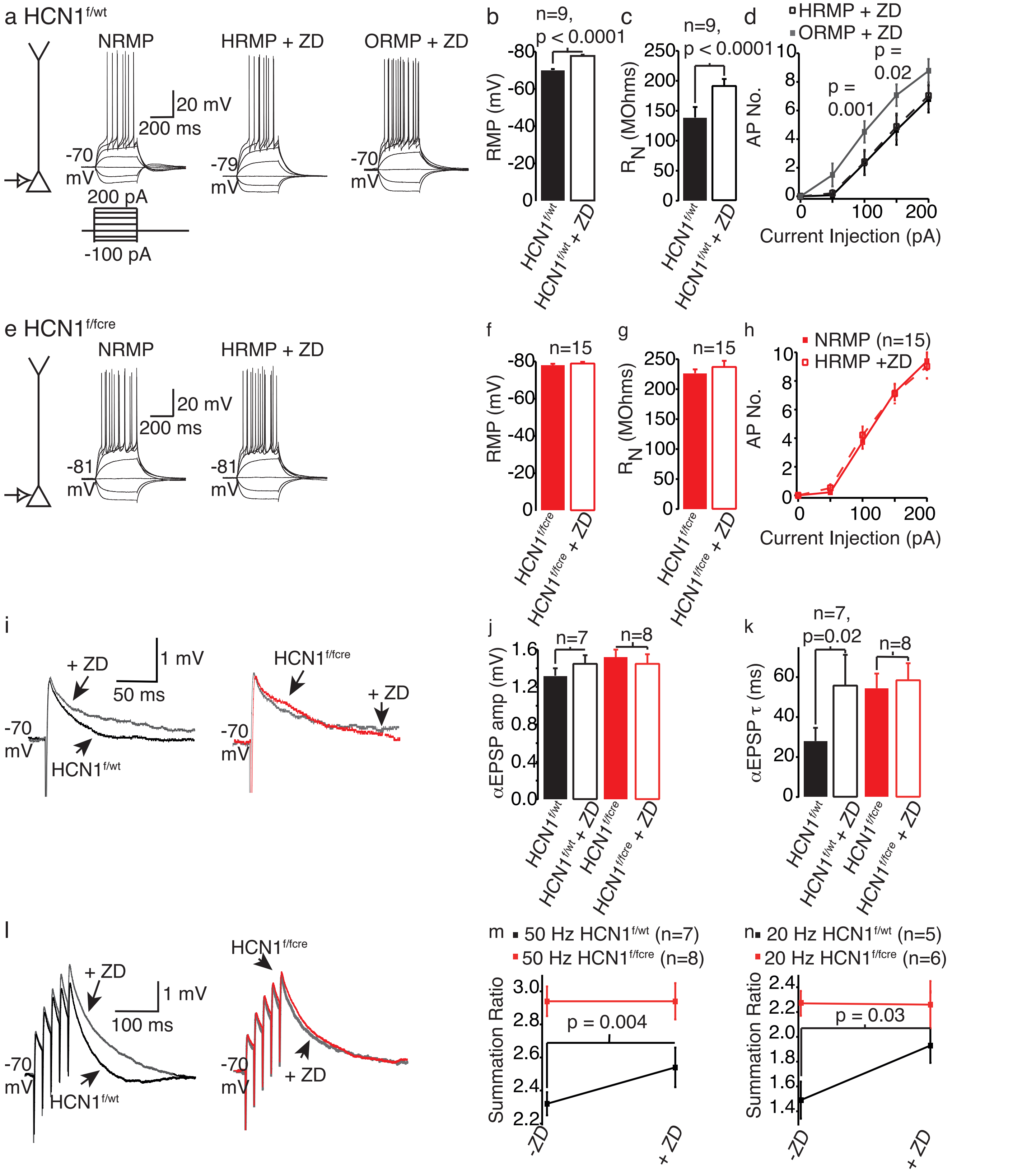

Supp Fig 2: The properties of HCN1$^{f/fcre}$ EC layer III neurons can be reproduced by pharmacological inhibition of HCN channels in wildtype neurons. a, e Example recordings obtained from HCN1$^{f/wt}$ and HCN1$^{f/fcre}$ neurons respectively when the step protocol shown in the schematic was applied before and after ZD7288 (ZD; 15 µM). In HCN1$^{f/wt}$ neurons, the RMP was hyperpolarized in the presence of ZD7288. Recordings in the presence of ZD7288 were, thus, made at the hyperpolarized RMP (HRMP) as well as by artificially injecting positive current to that of the original RMP (ORMP) value. The scale associated with the first trace in each panel applies to all traces within that panel. **b,c,d,e,f,g,h** Graphs illustrating the mean RMP, R_N and numbers of action potentials elicited by a given depolarizing pulse in HCN1$^{f/wt}$ and HCN1$^{f/fcre}$ neurons with and without ZD7288**. i, l** Representative traces of single and 50 Hz trains of αEPSPs in HCN1$^{f/wt}$ and HCN1$^{f/fcre}$ neurons in the absence and presence of ZD7288. The scale shown in each panel applies to all traces within that panel. **j, k, m, n** Bar Graphs depicting the average amplitude (amp) and decay time constant (τ) of individual αEPSPs as well as the mean 20 Hz and 50 Hz summation ratios of αEPSP trains in HCN1$^{f/wt}$ and HCN1$^{f/fcre}$ neurons prior to and following treatment with ZD7288. Significance (p) values are indicated when appropriate.

Huang et al., Supp Table 1

**Genotype and treatment**	**Amplitude (mV)**	**Decay time constant (ms)**	**Rise time constant (ms)**
$HCN1^{f/wt}$ - ZD	5.16 ± 0.27 (n=7)	14.66 ± 2.33 (n=7)	1.55 ± 0.10 (n=7)
$HCN1^{f/wt}$ +ZD	4.95 ± 0.25 (n=7)	14.81 ± 1.78 (n=7)	1.69 ± 0.06 (n=7)
$HCN1^{f/fcre}$ - ZD	5.27 ± 0.19 (n=9)	15.22 ± 0.71 (n=9)	1.52 ± 0.09 (n=9)
$HCN1^{f/fcre}$ + ZD	5.28 ± 0.19 (n=9)	15.91 ± 0.56 (n=9)	1.51 ± 0.08 (n=9)

Supp Table 1: **Spontaneous mEPSC characteristics in $HCN1^{f/wt}$ and $HCN1^{f/fcre}$ with and without ZD7288**. A comparison of mEPSC amplitude and kinetics obtained from $HCN1^{f/wt}$ and $HCN1^{f/fcre}$ EC layer III neurons in the absence and presence of the HCN channel inhibitor, ZD7288 (15 μM). In all experiments, treatment with ZD7288 was for 15 min only. In addition, to limit the effects of post-synaptic HCN channels on mEPSCs, the internal pipette solution for recording mEPSCs contained 15 μM ZD7288 (see **Methods**). The numbers of observations are indicated in parenthesis.

Genotype and Treatment	Control Amplitude (mV)	Amplitude following 10 Hz stimulation (mV)	Control Decay time constant (ms)	Decay time constant following 10 Hz stimulation (ms)
$HCN1^{f/wt}$ alone	0.18 ± 0.02 mV (n=9)	0.20 ± 0.02 mV (n=9)	20.64 ± 2.12 ms (n=9)	20.83 ± 1.66 ms (n=9)
$HCN1^{f/fcre}$ alone	0.21 ± 0.01 mV (n=9)	0.19 ± 0.02 mV (n=9)	34.40 ± 3.90 ms (n=9)	31.81 ± 4.74 ms (n=9)
$HCN1^{+/+}$ alone	0.21 ± 0.02 mV (n=6)	0.22 ± 0.02 mV (n=6)	21.10 ± 4.05 ms (n=6)	22.92 ± 4.29 ms (n=6)
$HCN1^{-/-}$ alone	0.24 ± 0.01 mV (n=6)	0.26 ± 0.02 mV (n=6)	64.76 ± 13.23 ms (n=6)	49.40 ± 12.89 ms (n=6)
Wt before and after ZD7288	0.22 ± 0.02 mV (n=13)	0.28 ± 0.02 mV (n=13)*	20.45 ± 3.66 ms (n=6)	38.32 ± 9.44 ms (n=6)*

Supp Table 2: Field EPSP characteristics before and after 10 Hz extracellular stimulation following FM1-43 loading into synaptic terminals. Comparison of the field EPSP amplitude and decay time constants in $HCN1^{f/wt}$, $HCN1^{f/fcre}$, $HCN1^{+/+}$ and $HCN1^{-/-}$ slices before and after 10 Hz extracellular stimulation when FM1-43 dye had been loaded into synaptic terminals (see **Methods**; Fig 3). In addition, for experiments in which the effects of 15 μM ZD7288 on synchronous release from wildtype (Wt) boutons was assessed (Fig 4), the amplitude and decay time constants of the field EPSPs prior to application of ZD7288 and after 20 min application of the compound (i.e. after the 10 Hz stimulation; see Fig 4) were measured. The number of observations for each group are indicated in parenthesis. Asterisks indicate significance at $p < 0.05$.

Huang et al., Supp Table 3

	Normalised Fluorescence – HCN1 null vs HCN1 wildtype slices (Fig 3e(i))	Comparison of fluorescence during 1.5 Hz stimulation between HCN1 null and HCN1 wildtype slices if fluorescence is normalised to that just prior to stimulation (Fig 3e(ii))	Normalised Fluorescence –wildtype slices vs wildtype slices treated with ZD7288 (Fig 4c(i))	Comparison of fluorescence during 1.5 Hz stimulation between wildtype slices and wildtype slice treated with ZD7288 if fluorescence is normalised to that just prior to stimulation (Fig 4c(ii))
Degrees of Freedom	22	12	22	12
Significance between groups	p < 0.0001*	p = 0.0198*	P < 0.0001*	p = 0.0059*
Baseline Time t=0 (min)	p = 0.1745	n/a	p < 0.0001	n/a
Baseline Time t=1 (min)	p > 0.9999	n/a	p > 0.9999	n/a
Baseline Time t=2 (min)	p = 0.0118*	n/a	p > 0.9999	n/a
Baseline Time t=3 (min)	p = 0.0003*	n/a	p > 0.9999	n/a
Baseline Time t=4 (min)	p = 0.0026*	n/a	p = 0.307	n/a
Baseline Time t=5 (min)	p < 0.0001*	n/a	p = 0.9692	n/a

Baseline Time t=6 (min)	p = 0.0001*	n/a	p > 0.9999	n/a
Baseline Time t=7 (min)	p = 0.0003*	n/a	p = 0.3888	n/a
Baseline Time t=8 (min)	p = 0.0002*	n/a	p > 0.9999	n/a
Baseline Time t=9 (min)	p > 0.9999	n/a	p > 0.9999	n/a
Baseline Time t=10 (min)	p > 0.9999	n/a	p > 0.9999	n/a
1.5 Hz stimulation, Time t=10.33 (min)	p > 0.9999	p > 0.9999	p > 0.9999	p > 0.9999
1.5 Hz Stimulation, Time t=10.67(min)	p = 0.0005*	p > 0.9999	p = 0.6326	p > 0.9999
1.5 Hz Stimulation, Time t=11 (min)	p < 0.0001*	p > 0.9999	p = 0.3742	p > 0.9999
1.5 Hz Stimulation, Time t=11.33 (min)	p < 0.0001*	p > 0.9999	p = 0.0002*	p = 0.0612
1.5 Hz Stimulation, Time t=11.67 (min)	p = 0.0018*	p > 0.9999	p = 0.0048*	p = 0.0195*
1.5 Hz Stimulation, Time t=12 (min)	p < 0.0001*	p > 0.9999	p = 0.0001*	p = 0.0020*
1.5 Hz Stimulation, Time t=12.33 (min)	p = 0.0166*	p > 0.9999	p = 0.0257*	p = 0.1556
1.5 Hz Stimulation,	p = 0.0378*	p > 0.9999	p = 0.0215*	p = 0.0396*

Time t=12.67 (min)				
1.5 Hz Stimulation, Time t=13 (min)	p = 0.0007*	p = 0.2721	p = 0.0002*	p = 0.0013*
1.5 Hz Stimulation, Time t=13.33 (min)	p = 0.0003*	p = 0.1588	p = 0.0001*	p = 0.0008*
1.5 Hz Stimulation, Time t=13.67 (min)	p = 0.0002*	p = 0.1931	p = 0.0003*	p = 0.0040*
1.5 Hz Stimulation, Time t=14 (min)	p < 0.0001*	p = 0.0189*	p = 0.0001*	p = 0.0008*

Supp Table 3: Comparison of FM1-43 de-staining in the absence and presence of HCN1 channels. A two-way ANOVA was used to determine the significance (p) values between slices in which HCN1 channels were present (wildtype, n=15) or absence (HCN1 null (n=15) or treated with ZD7288 (n=13). Subsequently unpaired t-tests with the p value adjusted for multiple comparisons using the Bonferroni-Dunn Method was utilised. $p < 0.05$ was considered to be significant and denoted using an asterisk.

Huang et al., Supp Table 4

$1/\tau$ range (s^{-1})	HCN1 null slices (n=15) vs HCN1 wildtype slices (n=15)	Wildtype vs Wildtype slices treated with ZD7288
0 – 0.002	p = 0.261	p = 0.0088*
0.002 – 0.004	p = 0.004*	p < 0.0001*
0.004 – 0.006	p = 0.187	p = 0.0011*
0.006 – 0.008	p < 0.0001*	p < 0.0001*
0.008 – 0.01	p > 0.9999	p > 0.9999
0.01 – 0.012	p > 0.9999	p > 0.9999
0.012 – 0.014	p = 0.8954	p > 0.9999
0.014 – 0.016	p = 0.0077*	p < 0.0001*
0.016 – 0.018	p = 0.2189	p = 0.0165*
0.018 – 0.020	p > 0.9999	p = 0.0055*
0.020 – 0.022	p > 0.9999	p = 0.0319*

Supp Table 4: Comparison of decay fitting time constant values. Table shows the p values obtained using two-tailed Mann-Whitney U tests with the p value adjusted for multiple comparisons using a Bonferroni Constant for the decay fitting time constant ranges showed in **Fig 3f** and **Fig 4d**. $p < 0.05$ was considered to be significant and is indicated by an asterisk (*).
